# Isolated pulmonary valve endocarditis treated with percutaneous aspiration: a case report and literature overview

**DOI:** 10.1186/s12872-025-05169-7

**Published:** 2025-09-29

**Authors:** Gabriel Roberto Lopez-Mora, Carlos Enrique Vesga-Reyes, Maria Juliana Reyes-Cardona, Jorge Alexander Zambrano-Franco, Jairo Sanchez-Blanco, Pastor Olaya, Miller Giraldo-Sandoval, Camilo Andres Calderon-Miranda

**Affiliations:** 1 Facultad de Ciencias de la Salud, Universidad Icesi, Calle 18 No. 122-135, Cali, 760031 Colombia; 2 Departamento de Medicina Interna, Fundación Valle del Lili, Carrera 98 No. 18-49, Cali, 760032 Colombia; 3 Departamento de Cardiología, Fundación Valle del Lili, Carrera 98 No. 18-49, Cali, 760032 Colombia; 4 Unidad de Intervencionismo Vascular, Fundación Valle del Lili, Carrera 98 No. 18-49, Cali, 760032 Colombia

**Keywords:** Bacterial endocarditis, Pulmonary valve, Intensive care unit, Ventricular septal defect, Vegetation aspiration, Methicillin-Resistant staphylococcus aureus, Septic shock

## Abstract

**Background:**

Pulmonary valve infective endocarditis (PVIE) is a rare condition, accounting for a small proportion of right-sided endocarditis cases. It poses unique diagnostic and therapeutic challenges and requires a multidisciplinary approach.

**Case presentation:**

We present the case of a 34-year-old male with obesity and a congenital ventricular septal defect, admitted to the intensive care unit for septic shock secondary to methicillin-resistant Staphylococcus aureus (MRSA) and multidrug-resistant Klebsiella pneumoniae. Transesophageal echocardiography revealed two large vegetations on the pulmonary valve. Due to prohibitive surgical risk, percutaneous aspiration was performed using the 16 Fr Lightning Flash™ system, achieving partial vegetation reduction. Despite procedural success, the patient died from refractory septic shock.

**Discussion:**

PVIE is associated with congenital heart defects, intravenous drug use and intracardiac devices. Diagnosis is often delayed due to nonspecific clinical features. Management includes prolonged antibiotic therapy, with surgery or minimally invasive interventions reserved for severe cases. Percutaneous vegetation aspiration, though rarely reported, offers a minimally invasive alternative for patients unfit for surgery. This case highlights its technical feasibility. While promising, further evidence is needed to assess outcomes and long-term efficacy.

**Supplementary Information:**

The online version contains supplementary material available at 10.1186/s12872-025-05169-7.

##  Background

Right-sided infective endocarditis (IE) represents approximately 10% of all IE cases, with predominant involvement of the tricuspid valve (90%). In contrast, infective endocarditis of the pulmonary valve (PVIE) is exceptionally rare, comprising only a small fraction of these cases. Its infrequency contributes to diagnostic delays and poses distinct therapeutic challenges. PVIE is most commonly associated with congenital heart defects, intravenous drug use, and intracardiac devices [1]. Cardiovascular imaging, particularly transesophageal echocardiography, plays a pivotal role in the diagnosis and characterization of this condition. Although the mainstay of treatment is prolonged antibiotic therapy and surgery in selected cases [2], percutaneous interventions such as vegetation aspiration have emerged as a potential alternative for patients who are not candidates for surgery. However, this technique remains scarcely documented in the medical literature.

We present the case of a 34-year-old male patient admitted for septic shock and pneumonia, found to have isolated PVIE and a ventricular septal defect (VSD), with contraindications for surgery.

##  Case presentation

A 34-year-old male with class III obesity (BMI: 42) and a childhood history of a heart murmur —without other known diagnoses— was admitted to the emergency department with one week of fever, productive cough and dyspnea. On admission, he was somnolent and diaphoretic, with signs of respiratory distress. Blood pressure was 104/43 mmHg, heart rate 129 beats per minute, respiratory rate 40 per minute, oxygen saturation 90% with a non-rebreathing mask. On auscultation, crackles and rhonchi were heard in both lung fields, in the context of the emergency department, no murmurs were reported. Abdominal and extremity examinations were unremarkable. The patient showed no evident focal neurological deficits.

Initial management for suspected septic shock and hypoxemic respiratory failure included invasive mechanical ventilation, fluid and dual vasopressor support and broad-spectrum antibiotics (meropenem plus vancomycin).

Laboratories revealed leukocytosis with marked neutrophilia and elevation of C-reactive protein, thrombocytopenia, elevated creatinine and blood urea nitrogen, hyponatremia and mild hypokalemia, direct hyperbilirubinemia suggesting sepsis-related hepatopathy, hyperlactatemia, severe hypoxemia and mixed (metabolic and respiratory) acidosis (Table 1). Blood cultures were positive for methicillin-resistant Staphylococcus aureus (MRSA).

**Table 1:** Laboratories on admission. *FiO2 *Fraction of inspired oxygen, *PO2* Partial pressure of oxygen, *PCO2* Partial pressure of carbon dioxide, *HCO3* Bicarbonate, *BE* Base excess

Test	Patient’s value	Reference value	Unit
Leucocyte count	25,720	3980–10,040	/µL
Neutrophil count	20,450	1560–6130	/µL
Lymphocyte count	2200	1000–4800	/uL
Hemoglobin	12.9	12-15.7	g/dL
Platelets	144,000	182,000–369,000	/µL
C-Reactive protein	54.07	0-0.5	mg/dL
Creatinine	3.6	0.67–1.17	mg/dL
Blood urea nitrogen	60.5	6–20	mg/dL
Total Bilirubin	3.15	0.14–1.4	mg/dL
Direct Bilirubin	2.99	0.09–0.3	mg/dL
Indirect Bilirubin	0.16	0.05–1.1	mg/dL
Sodium	123	135–145	mmol/L
Potassium	3.3	3.5–5.1	mmol/L
Lactate	2.5	0.5–2.2	mmol/L
COVID-19 Antigen	Negative		
HIV I – II Antibodies	Negative		
Arterial blood gases			
pH	7.20	7.35–7.45	
FiO2	1		
PO2	80	83–108	mmHg
PO2/FiO2 ratio	80		mmHg
PCO2	45	35–48	mmHg
HCO3	17.6	21–28	mmol/L
BE	−10.4		mmol/L

**Fig. 1:**
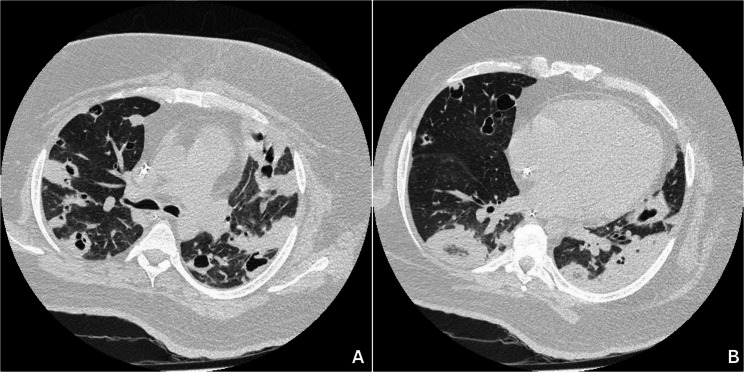
Chest computed tomography. Multiple centrally distributed peribronchovascular consolidations, many with central cavitation, irregular internal septations and dense material. Multiple bilateral pulmonary nodules (<8 mm) with random distribution. No pleural effusion is observed. Right paratracheal lymph nodes appear reactive

A chest computed tomography (CT) scan (Fig. 1) revealed multiple peribronchovascular consolidations, predominantly central in distribution, most of which exhibited central cavitation, with some showing irregular septations and dense internal material. A focal consolidation with a reverse halo sign was noted in the posterior basal segment of the right lower lobe. Additionally, multiple randomly distributed bilateral pulmonary nodules, all measuring less than 8 mm, were identified. There was no evidence of pleural effusion. Right paratracheal lymph nodes appeared reactive.

**Fig. 2:**
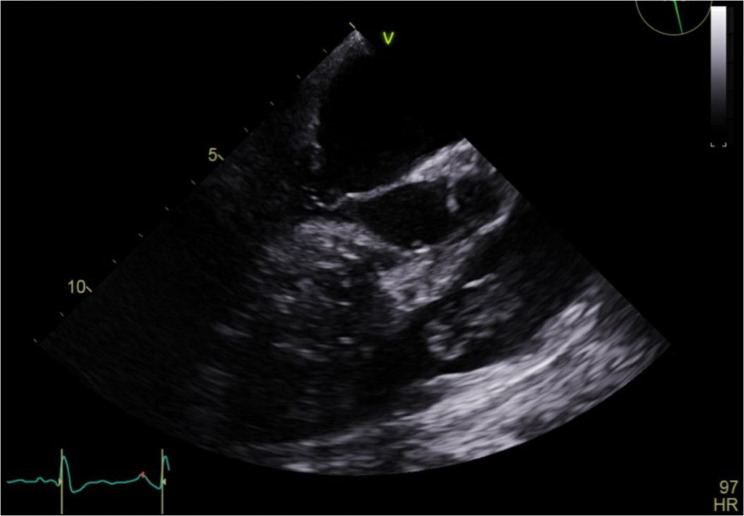
Transesophageal echocardiogram. Mid-esophageal short-axis. 2.0 × 0.8 cm mass suggestive of a vegetation adhered to the pulmonary valve. Mildly dilated right ventricle

**Fig. 3:**
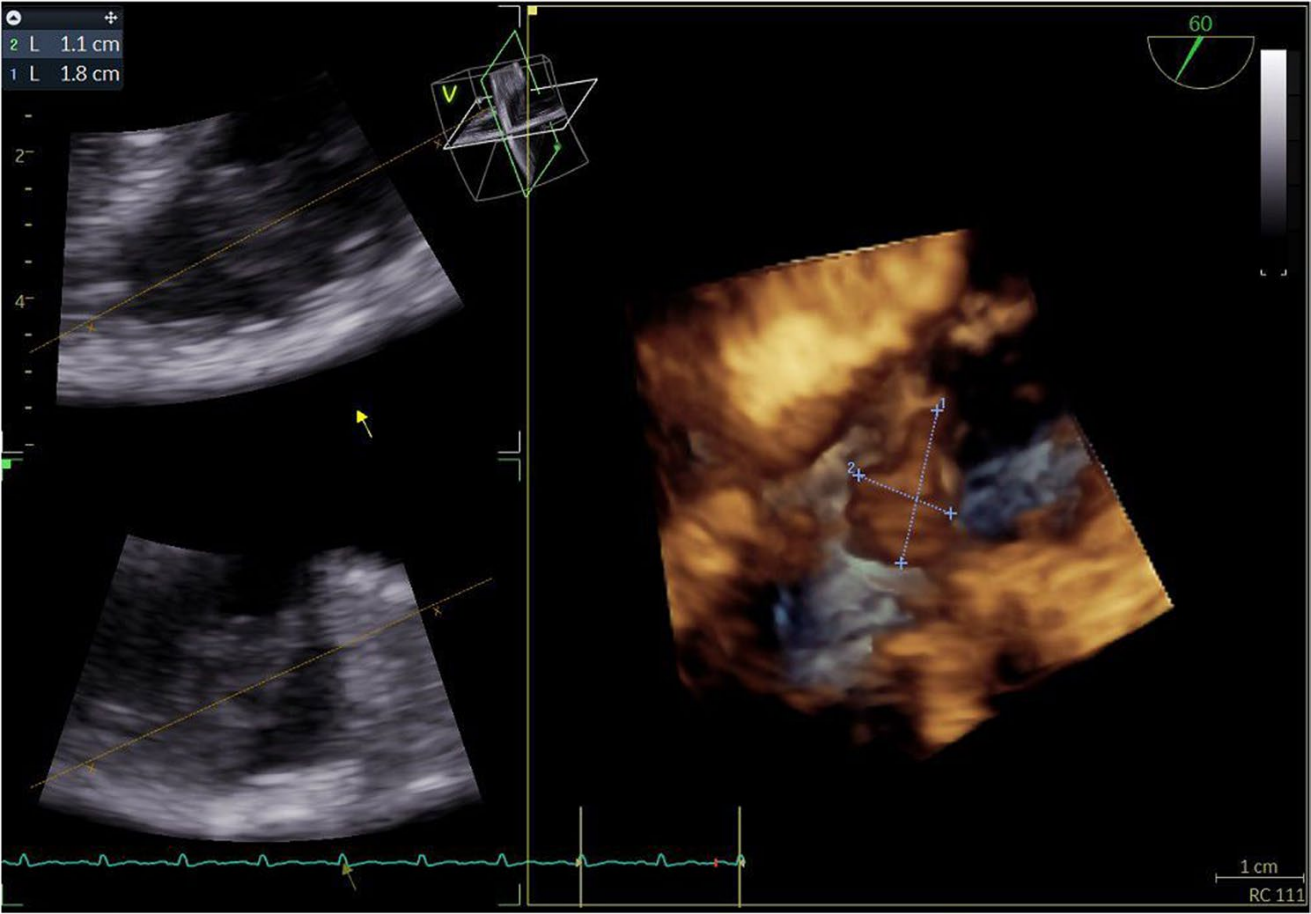
Transesophageal echocardiogram. 60º. 3D reconstruction of the vegetation on the pulmonary valve

The patient clinically deteriorated in the following 24 h, requiring deep sedation, neuromuscular blockade, and prone positioning due to refractory hypoxemia. Antibiotic therapy was adjusted to ceftaroline + daptomycin based on the blood cultures results and clinical worsening. A transthoracic echocardiogram (TTE) was attempted; however, image quality was significantly limited due to a poor acoustic window, likely related to the patient’s body habitus (class III obesity). Given the high clinical suspicion of infective endocarditis based on persistent bacteremia, congenital heart disease, and clinical deterioration, we proceeded with transesophageal echocardiography (TEE) to obtain better visualization of the cardiac structures. It revealed preserved left ventricular systolic function with an ejection fraction of 60–65%. The right heart chambers were mildly dilated. A restrictive perimembranous VSD was identified, with a left-to-right shunt demonstrating a peak velocity of 4 m/s and a peak gradient of 64 mmHg. Two vegetations were visualized on the PV, measuring 2.0 × 0.8 cm and 1.7 × 0.9 cm. Trivial to mild pulmonary regurgitation (PR) was identified. No involvement of other valves was observed (Figs. 2 and 3, See Supplementary file Video 1).

A diagnosis of isolated PVIE was established, supported by the presence of vegetations, bacteremia, and congenital heart disease as a predisposing factor. Due to worsening hemodynamic instability, surgical intervention was deemed high-risk and therefore contraindicated.

Despite appropriate antibiotic coverage, the patient developed persistent MRSA bacteremia, continued to require invasive mechanical ventilation and vasopressor support, and was started on continuous renal replacement therapy (CRRT). The multidisciplinary Heart Team opted for percutaneous vegetation aspiration to reduce the burden of infection.

The procedure was performed with a large-caliber suction equipment (16 Fr Lightning Flash ™ (Penumbra, Inc.) aspiration system). The Penumbra system was selected as it was the only aspiration system available in our institution at the time.

**Fig. 4:**
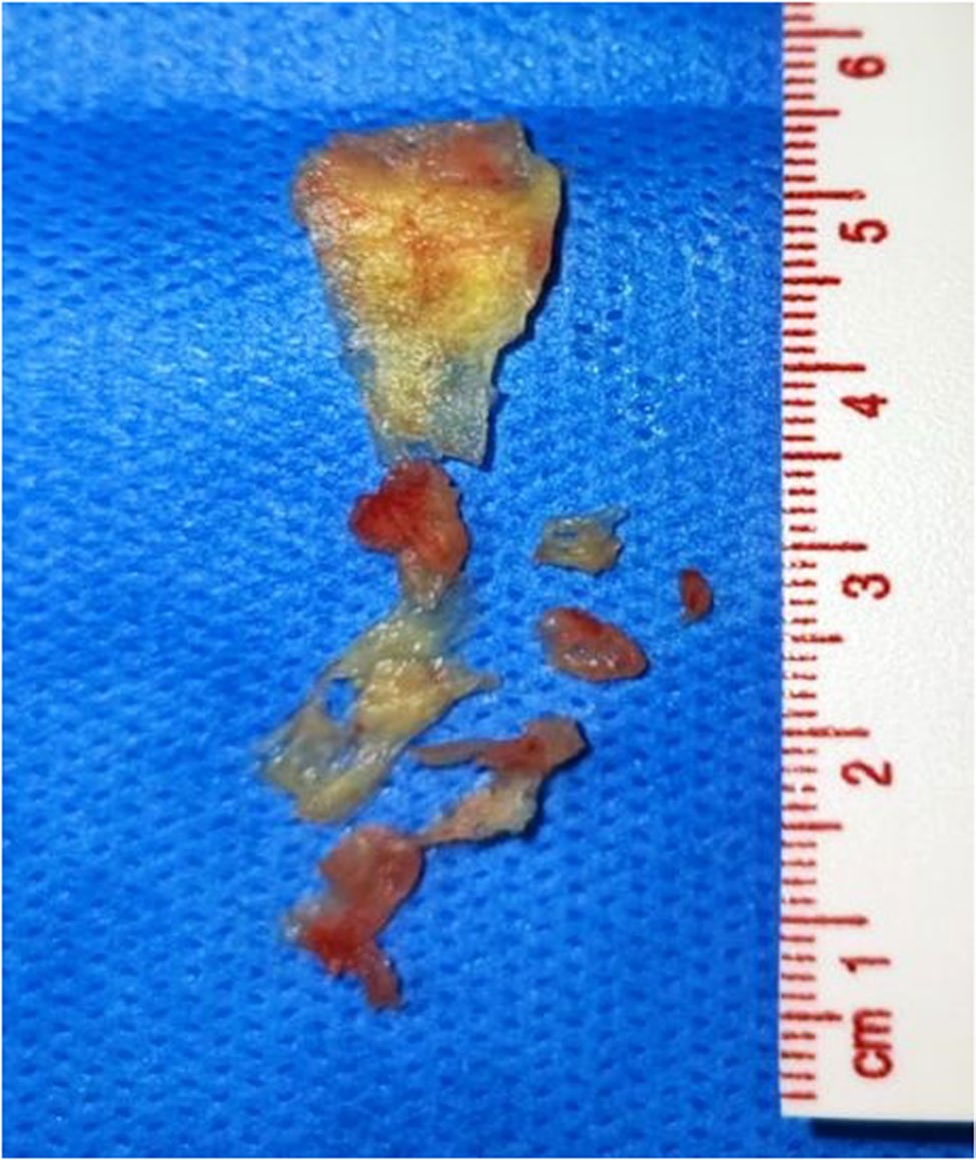
Yellowish material aspirated from the pulmonary valve

**Fig. 5:**
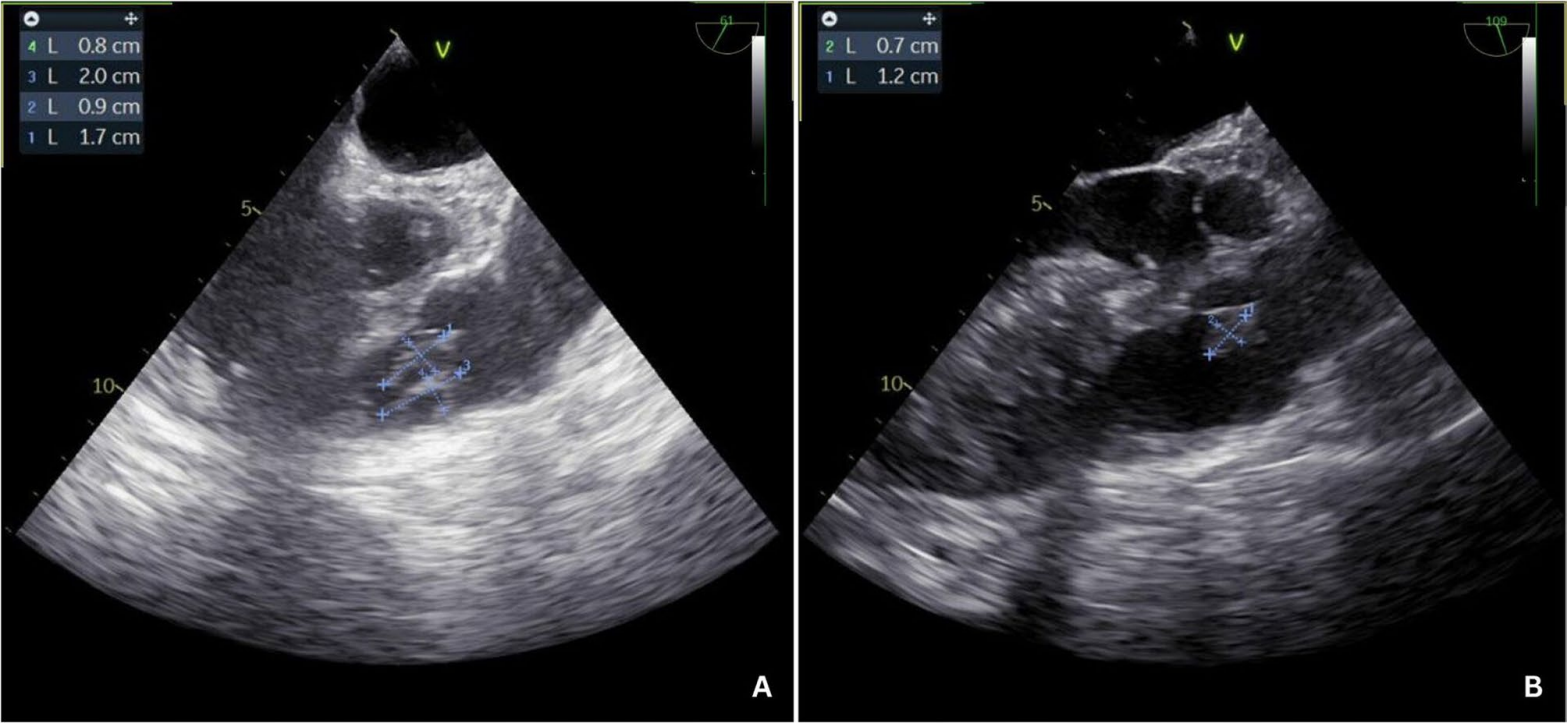
Transesophageal echocardiogram (TEE). A. Pre-procedural TEE. 61º. Two intracardiac masses suggestive of vegetations adhered to the pulmonary valve measuring 2.0 x 0.8 cm and 1.7 x 0.9 cm. B. Post-procedural TEE. 109º. One residual vegetation adhered to the pulmonary valve measuring 1.2 x 0.7 cm

Under general anesthesia and TEE guidance, right femoral vein access was obtained, and the catheter was advanced to the right ventricle. Once the catheter position was confirmed, aspiration of the vegetations was performed (See Supplementary files Video 2 and Video 3), retrieving yellowish material (Fig. 4). Post-procedural TEE confirmed significant reduction in the size of the vegetations and trivial to mild PR. The residual vegetation measured approximately 0.7 × 1.2 cm (Fig. 5).

The patient’s hemoglobin dropped progressively despite no evidence of bleeding. This decline can be explained by his critical condition, prolonged ICU stay, CRRT requirement, organ dysfunction, and intensive supportive therapies, all of which can contribute to worsening anemia even without overt blood loss. Pre-procedural hemoglobin was 7.3 g/dL; post-procedural hemoglobin dropped to 6.5 g/dL, prompting the transfusion of three units of red blood cells, which increased hemoglobin to 8.3 g/dL.

**Fig. 6:**
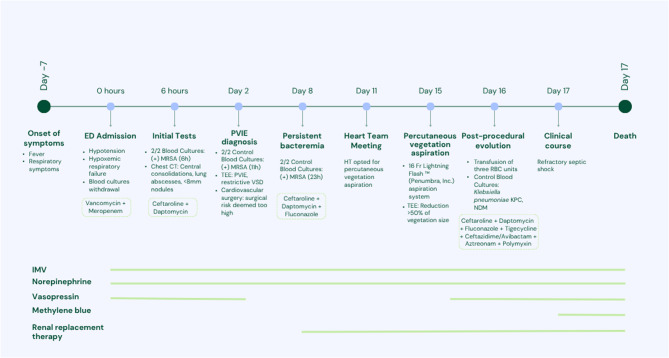
Timeline of events

Despite technical success, the patient remained critically ill. He developed profound shock requiring high-dose vasopressors, inotropic support, and renal replacement therapy. He continued to have recurrent fever and elevated inflammatory markers. Subsequent blood cultures revealed multidrug-resistant Klebsiella pneumoniae harboring Klebsiella pneumoniae carbapenemase (KPC) and New Delhi metallo-beta-lactamase (NDM) resistance genes. The antibiotic regimen was expanded to include polymyxin, aztreonam, ceftazidime-avibactam, and tigecycline. Despite aggressive therapy, the patient remained in critical condition and died two days later. Vegetation culture was positive for Klebsiella pneumoniae, with both Carba NP and ESBL positivity. The definitive culture report became available after the patient’s death. The vegetation was not sent for pathological examination and no pathological autopsy was performed. The timeline of events is summarized in Fig. 6.

##  Discussion and conclusions

Right-sided IE accounts for approximately 10% of all IE cases, with the tricuspid valve involved in up to 90% of these. PV involvement represents the remaining 10% [1]. Isolated PVIE —without other valve involvement—is even rarer. Key risk factors include intravenous drug use, congenital right-sided heart defects, alcohol abuse, male sex, and the use of intravascular medical devices such as central venous catheters or cardiac pacing leads [2, 3].

*Staphylococcus aureus* is the most common causative organism (70%), followed by coagulase-negative staphylococci (5–30%) and group B streptococci [4–6].

Fever is the most common symptom, present in up to 90% of cases, often accompanied by chills, anorexia, weight loss, malaise, headache, myalgias, arthralgias, night sweats, abdominal pain, and dyspnea [7]. Septic pulmonary emboli frequently cause respiratory symptoms, often mimicking respiratory tract infections. If diagnosis is delayed, complications may include pulmonary infarction, empyema, lung abscesses, and pleural effusion [2, 8].

In our case, the diagnosis was supported by positive blood cultures and extensive multilobar infiltrates on imaging. While the 2023 Duke–International Society for Cardiovascular Infectious Diseases (ISCVID) criteria are applicable, they were primarily developed for left-sided IE and may be less sensitive in right-sided cases, as signs such as murmurs, peripheral emboli, and immunologic phenomena may be absent [9]. In our case a heart murmur was not noted on initial or subsequent examinations, likely because the patient’s critical condition and the urgency of care made it difficult to properly assess for murmurs. However, given the peak velocity of 4 m/s, it is highly probable that a murmur was present but missed under these circumstances.

Echocardiography is a key diagnostic modality, though its performance in isolated PVIE is not well documented. In our case, TEE was chosen due to high clinical suspicion.

Empiric treatment often includes anti-staphylococcal beta-lactams and aminoglycosides [10]. Given our patient’s critical condition, broader antimicrobial coverage was initiated.

Antibiotic therapy usually spans 4–6 weeks but should be adjusted based on the isolated organism. Surgical treatment is indicated for persistent bacteremia despite one week of appropriate antibiotics, significant ventricular dysfunction, respiratory failure requiring mechanical ventilation, left-sided valve involvement, or large vegetations (> 20 mm) [10]. In our case, surgery was contraindicated due to hemodynamic instability.

Recently, percutaneous catheter-based aspiration has emerged as an alternative for selected patients. Current guidelines assign this approach a Class IIb, Level C recommendation [10]. Aspiration therapy is less invasive, does not require cardiopulmonary bypass, and has been successfully used for right atrial thrombi [11]. However, outcomes in right atrial pathology may not directly apply to right-sided IE due to differing disease mechanisms.

Percutaneous aspiration of tricuspid valve vegetations has been described in several case reports [12–14]. A 2022 systematic review and meta-analysis including 44 studies and 301 patients reported a procedural success rate of 89.2%, defined as at least a 50% reduction in vegetation size without major complications [15]. Aspiration therapy for isolated PVIE has been rarely reported in the literature, mainly in the context of pulmonary prosthetic valve endocarditis [16]. The decision to perform percutaneous aspiration of the vegetation was based on persistent bacteremia and increasing vegetation size despite appropriate antibiotic therapy, in a patient deemed ineligible for surgery due to prohibitive risk from hemodynamic instability and comorbidities. The expected benefits of the procedure included achieving infectious source control to facilitate clearance of bloodstream infection and resolution of septic shock and reducing the risk of septic emboli, which contributed to ongoing pulmonary and hemodynamic compromise. In critically ill patients not eligible for surgery, percutaneous aspiration represents a potential salvage option and has been described as a means to enhance antibiotic efficacy and mitigate embolic risk in the setting of refractory infection [17].

The AngioVac, FlowTriever, and Penumbra systems are minimally invasive devices used for the debulking of vegetations in right-sided infective endocarditis, each with distinct technical features, clinical outcomes, and complication profiles. The AngioVac system employs a large-bore (22 F) cannula connected to a veno-venous extracorporeal circuit, requiring dual vascular access (aspiration and reinfusion), which increases procedural complexity [18]. In a literature review [19], it demonstrated high procedural success, with an average of 87.9% across 9 studies involving 208 patients. However, complications such as hematomas, pericardial effusion, and worsening of valvular insufficiency have been reported [18].

The FlowTriever system uses a 24 F catheter with a T20 inner component and relies on manually generated aspiration via syringe. This eliminates the need for extracorporeal circulation, simplifying the procedural setup. Although it provides strong initial aspiration, the lack of continuous flow increases the risk of suction injury to cardiac structures. Outcome data specific to right-sided infective endocarditis are limited, but its single-site vascular access offers procedural advantages over AngioVac [19].

The Penumbra Lightning Flash system features a smaller 16 F aspiration catheter powered by an automated aspiration engine [20]. The catheter uses MaxID hypotube technology (allowing inner diameter similar to larger catheters with a lower profile). The Penumbra ENGINE (peristaltic pump) is used for continuous vacuum and advanced computer-assisted algorithms that allow dual clot detection capabilities (pressure differential and flow-based) for quicker clot and patent flow detection. A “Gallop Mode” is used for efficient thrombus removal, and the streamlined audio-visual cues provide real-time feedback to the physician, enhancing understanding of what is occurring at the catheter tip.- It is available in 80 cm, 100 cm, and 115 cm working lengths with HTORQ and XTORQ tip shapes for navigating complex anatomy. It requires direct contact with the vegetation to achieve effective suction, which increases the risk of distal embolization. This system has been evaluated in only two studies, demonstrating procedural success comparable to AngioVac; however, available data on complications and mortality remain limited [17].

Vascular access complications are less frequent with the Penumbra Lightning system compared to those associated with larger-bore aspiration systems [21]. However, most of the existing evidence comes from small case series. In our patient, significant bleeding occurred, as evidenced by the need for red blood cell transfusion.

In summary, the AngioVac system offers robust evidence for efficacy but presents logistical challenges. The FlowTriever system provides procedural simplicity, though technical limitations may exist. The Penumbra system, while less invasive, may be less effective for large vegetations due to its small bore. Each system has unique advantages and limitations, underscoring the need for further comparative studies to guide optimal device selection. See Table 2.

**Table 2:** Comparison of characteristics of different aspiration systems

Feature	Penumbra Lightning Flash	AngioVac	Inari FlowTriever
Mechanism	Computer-Assisted Vacuum Thrombectomy (CAVT): Utilizes a continuous, high-power vacuum pump (Penumbra ENGINE) combined with intelligent algorithms (pressure and flow-based) to differentiate between thrombus and flowing blood. When thrombus is detected, it maintains strong aspiration; when patent flow is detected, it reduces aspiration to minimize blood loss. Can also use a separator for denser clots.	Extracorporeal Venovenous Bypass and Suction: Employs a large-bore cannula with an expandable funnel tip that is advanced to the thrombus. Blood and thrombus are aspirated into an extracorporeal circuit, where the clot is filtered out, and the filtered blood is reinfused back into the patient.	Large-Bore Aspiration + Mechanical Thrombus Retrieval: Combines direct aspiration with mechanical removal. It uses a large-lumen aspiration catheter and self-expanding nitinol disks (Triever devices) that are deployed beyond the clot to engage and capture it, allowing for its mechanical extraction. Aspiration can be used concurrently.
Technical Aspects	Catheter Size: Generally midrange profile (e.g., 12-16F). Uses MaxID hypotube technology for larger inner diameter with a smaller outer profile. Catheter Design: Highly torqueable with a soft, atraumatic tip for navigation through tortuous pulmonary anatomy. Features visual and auditory feedback for clot detection. Ancillary Devices: Can use separator to fragment dense/organized thrombus. Anticoagulation: Requires systemic heparinization during the procedure.	Catheter Size: Large-bore (e.g., 22-26F cannula). Circuit Requirement: Requires an extracorporeal venovenous bypass circuit setup, involving a pump and filter. This necessitates larger access sites and potentially a longer setup time. Cannula Tip: Features an expandable funnel tip (balloon-actuated or selfexpanding) to enhance thrombus capture. Anticoagulation: Requires significant systemic anticoagulation (e.g., ACT >300 seconds) to prevent clotting in the extracorporeal circuit.	Catheter Size: Large-bore (e.g., 16F, 20F, 24F for Triever catheters and sheaths). Mechanical Component: Uses self-expanding nitinol mesh disks (Triever devices) that are deployed and retracted to capture and remove clot. Over-the-Wire System: Typically, an over-the-wire system, requiring a guidewire for navigation. Anticoagulation: Requires systemic heparinization during the procedure.
Advantages	Reduced Blood Loss: Intelligent aspiration minimizes aspiration of healthy blood, leading to lower estimated blood loss compared to non-intelligent high-power aspiration. Efficiency: Designed for rapid and efficient clot removal, potentially shortening procedure times. Trackability: Optimized catheter design allows for good navigability through complex pulmonary arteries. Less Invasive: Often avoids the need for full extracorporeal circulation, reducing associated risks.	Large Clot Burden Removal: Highly effective for aspirating large volumes of fresh, soft thrombus, including those in the right heart or IVC. No Hemolysis: The extracorporeal circuit filters the blood, preventing hemolysis often associated with rheolytic thrombectomy devices. Maintains Hemodynamics: Reinfusion of blood minimizes blood loss and helps maintain hemodynamic stability during aspiration.	Mechanical Thrombus Capture: The nitinol disks effectively capture and remove large and often more organized/walladherent clots that pure aspiration might miss. Single-Session Treatment: Designed to remove significant clot burden in a single session, often without the need for thrombolytics. No Thrombolytics: Generally, does not require thrombolytic agents, reducing the risk of major bleeding associated with systemic thrombolysis. Rapid Hemodynamic Improvement: Known for achieving immediate improvements in hemodynamics and symptoms.
Disadvantages	Learning Curve: Intelligent aspiration may require some familiarity to optimize use. Effectiveness on Organized Clot: While separator can help, may be less effective on very dense, chronic, or highly organized thrombus compared to mechanical retrieval options. Cost: High-tech system may involve higher upfront costs.	Invasiveness: Requires larger vascular access sites due to large-bore cannulas and extracorporeal circuit, increasing the risk of access site complications. Resource Intensive: Requires specialized setup and personnel for extracorporeal circulation. Limited Steerability in PA Branches: The larger and stiffer cannula can be challenging to maneuver into smaller, more peripheral pulmonary artery branches, limiting its utility for distal emboli. Risk of Extracorporeal Circuit Complications: Risks include air embolism, filter clotting, and potential for greater systemic anticoagulation.	Larger Catheter Profile: Requires larger access sheaths (up to 24F), increasing the risk of vascular access site complications (e.g., hematoma, pseudoaneurysm). Potential for Vessel Trauma: The mechanical retrieval component (disks) carries a theoretical risk of vessel wall trauma, though clinical data suggests a good safety profile. Blood Loss: While designed for clot capture, aspiration can still lead to blood loss, though efforts are made to minimize it.

In this case, we successfully performed percutaneous aspiration using the 16 Fr Lightning Flash™ system (Penumbra, Inc.), which was the only option available at our institution at the time. Our center had prior experience with this device in the treatment of acute pulmonary embolism. The AngioVac system is not yet available in our country, while the FlowTriever system had received regulatory clearance in the country but was not accessible at our institution. Despite technical success, the patient ultimately succumbed to refractory septic shock.

There is a lack of evidence to guide the management of isolated PVIE in patients with high surgical risk. While vegetation aspiration using endovascular devices has shown technical feasibility, reports remain limited to individual cases. Our experience supports the potential utility of this minimally invasive approach in right-sided IE. However, further studies are needed to evaluate outcomes, safety, and long-term prognosis.

## Supplementary Information

Supplementary Material 1.

Supplementary Material 2.

Supplementary Material 3.

## Data Availability

No datasets were generated or analysed during the current study.

## Abbreviations

BMI: Body Mass Index
CT: Computed Tomography
IE: Infective Endocarditis
ISCVID: International Society for Cardiovascular Infectious Diseases
KPC: Klebsiella pneumoniae Carbapenemase
MRSA: Methicillin-Resistant *Staphylococcus aureus*
NDM: New Delhi Metallo-beta-lactamase
PR: Pulmonary regurgitation
PV: Pulmonary Valve
PVIE: Pulmonary Valve Infective Endocarditis
TEE: Transesophageal Echocardiography
TTE: Transthoracic Echocardiography
VSD: Ventricular Septal Defect
CRRT: Continuous Renal Replacement Therapy

## References

[1] Chahoud J, Sharif Yakan A, Saad H, Kanj SS. Right-sided infective endocarditis and pulmonary infiltrates. Cardiol Rev. 2016;24(5):230–7. 26501991 10.1097/CRD.0000000000000095

[2] Chowdhury MA, Moukarbel GV. Isolated pulmonary valve endocarditis. Cardiology. 2016;133(2):79–82. 26501696 10.1159/000440857

[3] Revilla A, López J, Villacorta E, Gómez I, Sevilla T, del Pozo MÁ, et al. Isolated Right-Sided valvular endocarditis in Non-Intravenous drug users. Revista Española de cardiología. (English Edition). 2008;61(12):1253–9. 10.1016/s1885-5857(09)60052-9 19080963

[4] Edmond JJ, Eykyn SJ, Smith LD. Community acquired staphylococcal pulmonary valve endocarditis in non-drug users: case report and review of the literature. Heart. 2001;86(6):e17-17. 11711482 10.1136/heart.86.6.e17 PMC1729999

[5] Hamza N, Ortiz J, Bonomo RA. Isolated pulmonic valve infective endocarditis: a persistent challenge. Infection. 2004;32(3):170–5. 15188078 10.1007/s15010-004-3022-3

[6] Mathew J, Addai T, Anand A, Morrobel A, Maheshwari P, Freels S. Clinical features, site of involvement, bacteriologic findings, and outcome of infective endocarditis in intravenous drug users. Arch Intern Med. 1995;155(15):1641–8. 7618988

[7] Yuan SM. Right-sided infective endocarditis: recent epidemiologic changes. Int J Clin Exp Med. 2014;7(1):199–218. 24482708 PMC3902260

[8] Akinosoglou K, Apostolakis E, Marangos M, Pasvol G. Native valve right sided infective endocarditis. Eur J Intern Med. 2013;24(6):510–9. 23369408 10.1016/j.ejim.2013.01.010

[9] Prendergast BD. Diagnostic criteria and problems in infective endocarditis. Heart. 2004;90(6):611–3. 15145855 10.1136/hrt.2003.029850 PMC1768277

[10] Delgado V, Ajmone Marsan N, de Waha S, Bonaros N, Brida M, Burri H, et al. 2023 ESC guidelines for the management of endocarditis. Eur Heart J. 2023;44(39):3948–4042. 37622656 10.1093/eurheartj/ehad193

[11] Nezami N, Latich I, Murali N, Ali R, Lin BA, ShervinRad M, et al. Right atrial and massive pulmonary artery mechanical thrombectomy under echocardiography guidance using the FlowTriever system. EJVES Short Rep. 2019;45:22–5. 31828229 10.1016/j.ejvssr.2019.10.001 PMC6888743

[12] Hammad TA, Abu-Omar Y, Shishehbor MH. Novel intracardiac echocardiography‐guided catheter‐based removal of inoperable tricuspid valve vegetation. Catheter Cardiovasc Interv. 2022;99(2):508–11. 34766706 10.1002/ccd.29999

[13] Bangalore S, Alviar CL, Vlahakis S, Keller N. Tricuspid valve vegetation debulking using the angiovac system. Catheter Cardiovasc Interv. 2021;98(3):E475–77. 10.1002/ccd.29519 33565679

[14] Almanfi A, Nabous I. Percutaneous aspiration of vegetation in tricuspid valve infective endocarditis. JACC Case Rep. 2022;4(18):1151–5. 36213887 10.1016/j.jaccas.2022.03.032 PMC9537088

[15] Mhanna M, Beran A, Al-Abdouh A, Jabri A, Sajdeya O, Al-Aaraj A, et al. Angiovac for vegetation debulking in right-sided infective endocarditis: a systematic review and meta-analysis. Curr Probl Cardiol. 2022;47(11):101353. 35961428 10.1016/j.cpcardiol.2022.101353

[16] Márquez DT, Jiménez-Valero S, Jurado-Román A, Galeote G, Meras-Colunga P, Aroca Á, et al. Life-saving combination of extracorporeal membrane oxygenation, percutaneous aspiration, and valvuloplasty in a pulmonary prosthetic valve endocarditis. Can J Cardiol. 2025;41(4):761–3. 39151561 10.1016/j.cjca.2024.08.267

[17] Marinacci LX, Sethi SS, Paras ML, El Sabbagh A, Secemsky EA, Sohail MR, et al. Percutaneous Mechanical Aspiration for Infective Endocarditis: Proceedings From an Inaugural Multidisciplinary Summit and Comprehensive Review. J Soc Cardiovasc Angiogr Interv. 2024;3(12):102283. 10.1016/j.jscai.2024.102283 PMC11725074 39807229

[18] Poliwoda SD, Durbach JR, Castro A, Herman J, Caltagirone C, Kurup A, et al. AngioVac system for infective endocarditis. Ann Card Anaesth. 2023;26(1):105–8. 36722599 10.4103/aca.aca_156_21 PMC9997473

[19] Wang W, Itagaki S, Egorova N. Minimally invasive procedures for right side infective endocarditis: a targeted literature review. Catheter Cardiovasc Interv. 2024;103(6):1050–61. 38363035 10.1002/ccd.30967

[20] Coutas T, Mannarino M, Ventura M, Fay J, Simões R, Mannarino G. Technical aspects of penumbra Indigo lightning flash system for mechanical thrombectomy of pulmonary embolism: A comprehensive review. J Endovasc Ther. 2024:15266028241266148. 10.1177/15266028241266148 39057920

[21] El Sabbagh A, Yucel E, Zlotnick D, Moriarty JM, Younes S, Hamid N, et al. Percutaneous mechanical aspiration in infective endocarditis: applications, technical considerations, and future directions. J Soc Cardiovasc Angiogr Interv. 2024;3(4):101269. 39130180 10.1016/j.jscai.2023.101269 PMC11307789

